# Markers of MEK inhibitor resistance in low-grade serous ovarian cancer: EGFR is a potential therapeutic target

**DOI:** 10.1186/s12935-019-0725-1

**Published:** 2019-01-08

**Authors:** Marta Llaurado Fernandez, Amy Dawson, Joshua Hoenisch, Hannah Kim, Sylvia Bamford, Clara Salamanca, Gabriel DiMattia, Trevor Shepherd, Mattia Cremona, Bryan Hennessy, Shawn Anderson, Stanislav Volik, Colin C. Collins, David G. Huntsman, Mark S. Carey

**Affiliations:** 1 Obstetrics and Gynecology, University of British Columbia, Vancouver, BC Canada; 2 Pathology and Laboratory Medicine, University of British Columbia, Vancouver, BC Canada; 3 Translational Ovarian Cancer Research Program, London Health Science Centre, London, ON Canada; 4 Oncology, University of Western Ontario, London, ON Canada; 5 Medical Oncology, Royal College of Surgeons in Ireland, Beaumont Hospital, Dublin, Ireland; 6 Laboratory for Advanced Genome Analysis, Vancouver Prostate Centre, Vancouver, BC Canada; 7 Molecular Oncology, British Columbia Cancer Agency, Vancouver, BC Canada; 8 Division of Gynecologic Oncology, Diamond Health Centre, 2775 Laurel St., 6th Floor, Vancouver, BC V5Z 1M9 Canada

**Keywords:** Ovarian cancer, MEK inhibitor, EGFR inhibitor, PKC-alpha, Predictive biomarkers

## Abstract

**Background:**

Although low-grade serous ovarian cancer (LGSC) is rare, case-fatality rates are high as most patients present with advanced disease and current cytotoxic therapies are not overly effective. Recognizing that these cancers may be driven by MAPK pathway activation, MEK inhibitors (MEKi) are being tested in clinical trials. LGSC respond to MEKi only in a subgroup of patients, so predictive biomarkers and better therapies will be needed.

**Methods:**

We evaluated a number of patient-derived LGSC cell lines, previously classified according to their MEKi sensitivity. Two cell lines were genomically compared against their matching tumors samples. MEKi-sensitive and MEKi-resistant lines were compared using whole exome sequencing and reverse phase protein array. Two treatment combinations targeting MEKi resistance markers were also evaluated using cell proliferation, cell viability, cell signaling, and drug synergism assays.

**Results:**

Low-grade serous ovarian cancer cell lines recapitulated the genomic aberrations from their matching tumor samples. We identified three potential predictive biomarkers that distinguish MEKi sensitive and resistant lines: *KRAS* mutation status, and EGFR and PKC-alpha protein expression. The biomarkers were validated in three newly developed LGSC cell lines. Sub-lethal combination of MEK and EGFR inhibition showed drug synergy and caused complete cell death in two of four MEKi-resistant cell lines tested.

**Conclusions:**

*KRAS* mutations and the protein expression of EGFR and PKC-alpha should be evaluated as predictive biomarkers in patients with LGSC treated with MEKi. Combination therapy using a MEKi with EGFR inhibition may represent a promising new therapy for patients with MEKi-resistant LGSC.

**Electronic supplementary material:**

The online version of this article (10.1186/s12935-019-0725-1) contains supplementary material, which is available to authorized users.

## Background

Each year in Canada and the United States, over 25,000 women are diagnosed with ovarian cancer [1, 2]. Low-grade serous ovarian cancer (LGSC) accounts for 5–10% of these cancers [3, 4], affecting approximately 2000 women per year. This rare form of ovarian cancer is often diagnosed in pre-menopausal women and frequently found in advanced stages. Although LGSC is considered to be a less aggressive subtype than other ovarian cancers, response rates to chemotherapy are low, ranging from 4 to 25% [5]. Consequently, long-term fatality rates are high with only 10–20% of women surviving 10 years after diagnosis [5, 6].

It is now recognized that LGSC has unique clinical, pathological, and molecular characteristics compared to other types of ovarian cancers, such as the high-grade serous ovarian carcinoma (HGSC) [7, 8]. Molecular studies performed on LGSC tumors revealed that mutations in the *TP53* gene are rare (8% in LGSC versus 96% in HGSC) [9, 10], and that expression of estrogen (ER) and progesterone (PR) receptors is frequently observed [11, 12]. LGSC is also characterized by activation of the mitogen-activated protein kinase (MAPK) pathway. Mutations affecting this pathway are seen in *KRAS* (20–40%), *NRAS* (7–26%) and *BRAF* (5–33%) genes [13–20]. Evidence of MAPK pathway activation in LGSC [21] led to a key clinical trial evaluating the efficacy of the MEK inhibitor (MEKi) selumetinib for the treatment of patients with advanced and/or recurrent LGSC (GOG-0239). The results from this trial, published in 2013, shown a 15% response rate and 65% disease stabilization [22]. A second clinical trial of the MEKi binimetinib (MILO trial, NCT01849874) was closed at the interim analysis in 2016, because it did not show the anticipated predefined benefits on progression-free survival (PFS). Despite these unexpected results, durable responses to binimetinib were observed in LGSC with MAPK pathway alterations [23]. Currently, an international randomized phase II/III clinical trial using the MEKi trametinib is ongoing (NCT02101788) and a translational research component to better understand the molecular mechanisms of MEKi efficacy is included.

To date, preclinical laboratory research in LGSC has been limited to tumor tissues. The low frequency and slow growth rate of these tumors have challenged the development of cell lines and animal xenograft models. In the past 5 years, our laboratory has successfully established a collection of patient-derived LGSC cell lines that are now available for pre-clinical drug testing. Previously, we evaluated the effects of four different MEKi (selumetinib, trametinib, binimetinib, refametinib) in eight advanced/recurrent LGSC cell lines. Our results indicated that there were substantial differences in cellular response and on-target drug efficacy between cell lines and drugs [24]. Encouraged by promising results from MEKi clinical trials in a subset of LGSC patients, we sought to identify biomarkers that could predict response to treatment using LGSC cell lines, by comparing the proteogenomic profiles of MEKi-sensitive (MEKi-Se) and MEKi-resistant (MEKi-Re) LGSC cell lines, and subsequently evaluating the potential therapeutic value of two proteins (EGFR and PKC-alpha) associated with MEKi resistance.

## Materials and methods

### Tumor samples and clinical information

Advanced or recurrent LGSC samples (tumor and ascites) were obtained from the OvCaRe gynecologic tumor bank (Vancouver General Hospital/British Columbia Cancer Agency (BCCA), and the John and Mary Knight Translational Ovarian Cancer Research Unit (London Regional Cancer Program, London, Ontario, Canada). Tumor bank protocols, cell line derivation, and all research relating to this study was approved by institutional human ethics review boards at BCCA (H14-02859), the University of British Columbia (UBC; R05-0119), and the University of Western Ontario (HSREB 12668E). Clinical information was extracted retrospectively from patient records. Tumor histology was confirmed by a gynecological pathologist.

### Patient-derived LGSC cell lines

Low-grade serous ovarian cancer patient-derived cell lines were established through continuous in vitro culture of patient material obtained through OvCaRe or the John and Mary Knight Translational Ovarian Cancer Research Unit (cell line iOvCa241) tumor banks. Cultures were established and maintained in M199:MCDB105 (1:1) media (Cat. No. M5017 and M6395, Sigma-Aldrich, Oakville, Ontario, Canada) supplemented with 10% defined fetal bovine serum (dFBS; Cat. No. SH30070.03, Hyclone, GE Life Sciences, Logan, UT, USA) maintained at 37 °C and 5% CO_2_. No immortalization methods were used. Doubling time of these cells ranged from 30 to 80 h, with an average of 47 h, reflecting the clinical slow growth rate of LGSC.

### Sample authentication (cell line, tumor, buffy coat)

Microsatellite analysis of short tandem repeats (STRs) was performed on LGSC cell lines and corresponding tumor and buffy coat samples for cell line authentication. STR analyses of 10 loci were performed by Genewiz Inc. (South Plainfield, NJ) (Data available upon request). STR results confirm that all LGSC cell lines and buffy coat samples match to corresponding tumor samples.

### Genome sequencing

*Whole exome sequencing (WES)*: Agilent SureSelect RNA Library All Exons v6 protocol was performed by Beijing Genome Institute, per manufacturer’s guidelines. Quality and quantity of post-capture libraries were assessed using an Agilent 2100 Bioanalyzer. Libraries were sequenced on an Illumina Hiseq 4000 (PE 100). *Copy number variation (CNV) analysis*: Data analysis was performed using Nexus Copy Number Discovery Edition Version 9.0 (BioDiscovery, Inc., El Segundo, CA). Samples were processed using the Nexus NGS functionality (BAM ngCGH) with the FASST2 segmentation. The log ratio thresholds for single copy gain and single copy loss were set at + 0.18 and − 0.18, respectively. The log ratio thresholds for gain of 2 or more copies and for a homozygous loss were set at + 0.6 and − 1.0, respectively. Tumor sample BAM files were processed with corresponding normal tissue BAM files. Probes were normalized to median. *Mutation analysis*: Sequence alignment and mutation calling were performed in Partek Flow environment (© 2017 Partek Inc). Sequence reads were aligned to GRCh38/hg38 human genome build using bwa 0.7.2. Variants were called using Strelka 1.0.15 for all cell lines except for VOA-1312 (lacking buffy coat sample). VOA-1312 variant calling was performed using LoFreq 2.1.3.a. The called variants were annotated using the wAnnovar software (reference obtained from: http://jmg.bmj.com/content/49/7/433.citation-tools). Annotated calls were then filtered to show only protein-changing SNVs that were present in cell line DNA at allele frequencies (AF) greater than 0.1 and coverage higher than 16×. For VOA-1312, all calls not present in dbSNP (version 138) were retained, while of the calls that were present in dbSNP, only calls with (average heterozygocity + aveHet standard error) < 0.1 were retained. These were additionally filtered using the same criteria as for the Strelka calls.

*Whole genome sequencing (WGS)*: Genomic data from LGSC tumors T7 and T11 were obtained from the personalized oncogenomics (POG) program at the BCCA. Methodology has been previously described in detail [25]. To summarize, genome and transcriptome libraries were sequenced on HiSeq instruments (Illumina, San Diego, California) using V3 or V4 chemistry and paired-end 150 or 125 base reads, respectively. Target depth was 80× coverage for the tumor genome and 40× for the normal genome.

### Cell proliferation assays

Assessment of MEKi sensitivity using trametinib (GSK1120212; Sellekchem, Cat. No. S2673) and selumetinib (AZD6244; Cat. No. S1008), were performed as previously described [24]. Cell proliferation was monitored using IncuCyte™ real-time imaging technology using a non-labeled monolayer confluence approach (Essen Biosciences, Ann Arbor, MI, USA). LGSC cell lines were plated at 15–20% confluence in 96-well plates. After 24 h, cells were treated once with DMSO (control) or differing drug concentrations [erlotinib alone (10 μM and 2.5 μM), in combination (10, 5, 2.5, 1.25, and 0.63 μM), high and low doses of MEKi treatment (1 μM and 0.5 μM selumetinib; 0.1 μM and 0.05 μM trametinib; doses for preclinical MEKi assays as previously published)] [24]. Trametinib and selumetinib were selected as the MEKi for combination treatments. These two drugs are most commonly used clinically for treating LGSC, and binimetinib may lack efficacy based on results from the MILO clinical trial (NCT01849874). Drug doses of selumetinib and trametinib were chosen based on IC50 results from our previous experiments [24]. Selected concentrations for these experiments are in keeping with steady state serum levels (selumetinib 2 μM and trametinib 30 nM) reported for these drugs in humans [26, 27]. Phase contrast images of cells were taken every 6-h for 4–5 days. Each condition was evaluated using four technical replicates and experiments were repeated for verification. Data analysis was performed using IncuCyte™ software. Statistical analyses using the t-test on the final time point values of each assay were performed to compare treatment conditions. Differences were considered significant at a p-value < 0.05.

### Cell viability assay

Cell viability was measured using MTS-Cell Titer 96R Aqueous Non-Radioactive Cell Proliferation Assay, following manufacturer recommendations (Cat. No. G5430, Promega, Madison, WI, USA) at endpoint of Incucyte™ proliferation assays. Treatment media was replaced with 100 μL of fresh media and 20 μL of MTS. Plates were incubated for 3.5 h at 37 °C in humidified 5% CO_2_. Absorbance at 490 nm was measured using a microplate reader (BioTek Epoch SN257811). Viability for each treatment was compared to DMSO treated cells. Wells were subsequently stained with crystal violet (CV) to determine residual cells after treatment. Statistical analysis using t-test were used to compare treatment conditions and differences were considered significant at a p-value < 0.05.

### IC50 determination

Erlotinib (Cat. No. S7786) were purchased from Selleck Chemicals (Houston, TX, USA). Dimethylsulfoxide (DMSO; Sigma, Cat. No. D2650) was purchased from Sigma-Aldrich (Oakville, Ontario, Canada). Cells were seeded in 96-well plates at 40–50% confluence and treated after 24 h with DMSO or a range of drug concentrations. The inhibitory concentration (IC50, representing 50% of total cell viability) was determined using crystal violet staining after 72 h drug treatment.

### Western blot analysis

Cell lysates were prepared according to previously published protocols [24], then 20 μg samples were separated on an 8% SDS-PAGE gel, transferred to nitrocellulose membranes and probed with primary antibodies including ERK1/2 (Millipore, Cat. No. 06-182), p-MAPK (p-ERK1/2, Cell Signaling, Cat. No. 4376S), MEK1/2 (Cell Signaling, Cat. No. 9122), p-MEK1/2 (Cell Signaling, Cat. No. 9154), PKC-alpha (Cell Signaling, Cat. No. 2056), EGFR (Santa Cruz, Cat. No. 71032), p-EGFR (Cell Signaling, Cat. No. 2234), PARP (Cell Signaling, Cat. No. 9542), and c-PARP (Cell Signaling, Cat. No. 9541S). Vinculin (V9131, Sigma) was used as a protein loading control. Horseradish peroxidase (HRP)-conjugated secondary antibodies (goat-anti-mouse or goat-anti-rabbit, Sigma Cat. No. A9917 and A0545) were used accordingly. Western blots were imaged using Immobilon HRP reagent (Cat. No. WBKLS0500, Millipore, Etobicoke, ON, Canada) and developed by autoradiograph.

### Reverse-phase protein array (RPPA) analysis

Reverse-phase protein array on whole tumor and cell line lysates was performed as previously described [28, 29]. Proteomic profiles of 8 LGSC cell lines, 2 MEKi-sensitive (VOA-1312, iOvCa241) and 6 MEKi-resistant (VOA-1056, VOA-3993; VOA-3448, VOA-3723; VOA-4627, VOA-4698), were analyzed. LGSC cells were treated for 24 h with 1 μL/mL DMSO or MEKi (trametinib 0.1 μM, selumetinib 1.0 μM) in biological triplicate as previously described [24, 30]. Antibodies (n = 91) against cell surface growth factor receptors, common signaling pathway proteins, steroid hormone receptors, and other proteins involved in proliferation and apoptosis were used (Additional file 1: Table S1). Data was analyzed using SPSS software (Version 20, Chicago, Illinois). Differentially expressed proteins between cell lines and treatment conditions were determined using the t-test [31]. The Mann–Whitney U test was used for proteins with non-normally distributed expression levels. False discovery rates were not calculated as putative markers were validated by western blot.

### shRNA-mediated knockdown of PKC-alpha expression (PRKCA gene)

shERWOOD-UltramiR shRNA lentiviral target gene set containing three *PRKCA* shRNA sequences and one non-target shRNA (Cat. No. TLHVU1401-5578) was purchased from transOMIC Technologies (Huntsville, AL). VOA-3723 and VOA-6406 were plated at 50% confluence in 6-well tissue culture dishes 24 h prior to lentiviral transduction. 199:105 media supplemented with 1% Hyclone dFBS and polybrene (2 µg/mL for VOA-3723, 0.5 µg/mL for VOA-6406) and lentivirus expressing non-targeting shRNA or *PRKCA* shRNA (multiplicity of infection [MOI] = 26 for VOA-3723, MOI = 1.5 for VOA-6406) in a total volume of 1.5 mL was added. After 24 h, cells were washed with PBS and complete media was added. Successful transduction was confirmed using confocal microscopy. After an additional 24-h recovery, transduced LGSC cells were selected and maintained using puromycin (1.0 µg/mL for VOA3723, 0.5 µg/mL for VOA6406).

### Drug synergy analysis

Cell proliferation, viability and crystal violet results from in vitro drug testing (single drug and drug combinations) were used to assess drug synergism using CompuSyn software (http://www.combosyn.com). This software is based on the median-effect principle and the combination index-isobologram theorem (Chou-Talalay) [32]. Drug doses (D) and effects (fa) were entered (non-constant ratios) for single drug doses and combinations, and combination indices (CI) were generated. The CI values quantitatively defined synergism (CI < 1), additive effect (CI = 1), and antagonism (CI > 1).

## Results

### Development and MEKi treatment evaluation of LGSC cell lines

Our laboratory previously established a collection of LGSC cell lines derived from patients with advanced/recurrent disease. Preclinical evaluation of four MEKi in eight different LGSC cell lines resulted in the identification of two distinct phenotypes: MEKi-sensitive (MEKi-Se) cell lines (n = 2), and MEKi-resistant (MEKi-Re) cell lines (n = 6). In this first study, MEKi drug concentrations and IC50 values were reported [24]. Recognizing the challenges using IC-50 values to assess drug efficacy in-vitro, we established a stringent definition of MEKi sensitivity/resistance recognizing that only 15% of patients with advanced/recurrent LGSC will show tumor experience regression when treated with a MEKi. Thus, we classified cell lines as MEKi-Se if a single dose of MEKi resulted in complete cell death over a period of 5 days. Alternatively, cell lines were considered MEKi-Re if they continued to proliferate (even despite some degree of inhibition) under the same treatment conditions. Continuing our previous work, we established three new LGSC cell lines from three independent patients (VOA-6406, VOA-8862, VOA-9164). Tumor cells from three other LGSC patients were also grown temporarily as primary cultures (VOA-6800, VOA-6857, VOA-7604). Using our previous classification criteria, two of these new lines were classified as MEKi-Se (VOA-9164 and VOA-8862), and one as MEKi-Re (VOA-6406—see Additional file 2: Figure S1). STR analysis confirmed unique microsatellite profiles for each of these lines, matching the profiles of the original tumor tissues from which they were derived (data available upon request).

### Genomic characterization of MEKi-Se and MEKi-Re LGSC cells

**Fig. 1:**
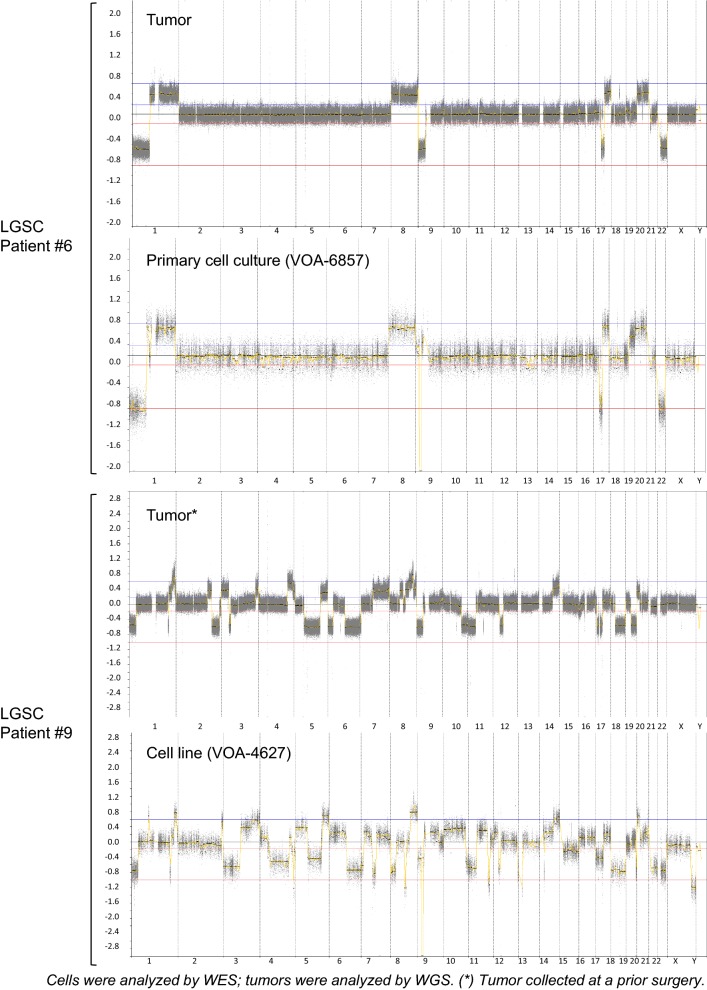
Comparison of genomic profiles between two LGSC cell cultures and their associated LGSC tumor samples. Each graph represents the copy-number (CN) changes detected per chromosome in each sample. Top graphs correspond to LGSC patient #6; CN changes detected in one of her recurrent tumor tissues was compared to the CN changes detected in the primary cell culture derived from this tissue. Bottom graphs correspond to the LGSC patient #9; CN changes detected in one of her recurrent tumor tissues was compared to the CN changes detected in the cell line established from a later recurrent tissue. High genomic profile correlation was observed between cells and tumors in both cases

WES was performed to characterize the genomic profiles of our LGSC cell lines and primary cultures. First, we compared the copy number profiles of two of our cell cultures (VOA-4627, VOA-6857) with those of their associated tumor samples (from WGS data). As shown in Fig. 1, the copy number variation profiles of the paired samples showed a very high degree of correlation. Of note, VOA-4627 line was derived from an ascites sample taken 2 years after the tumor sample collected previously at cytoreductive surgery.

Results from the WES analysis in our LGSC cell lines and primary cultures (n = 14) show variable levels of genomic aberration and non-synonymous mutations (NsMs), ranging from 1 to 66% total genome change and 24-111 mutation calls per cell line (Additional file 3: Table S2a). Deletion of Chr9p, including loss of *MTAP* and *CDKN2A* tumor suppressor genes, was found in all samples. As expected, *KRAS* and *NRAS* non-synonymous mutations were both found most frequently. Either mutation was present in 28.6% of all cell lines/cultures, and in 36.4% (*KRAS)* and 27.3% (*NRAS*) respectively when analyzed by patient (some cell lines were derived from the same patient at different times). *KRAS* and *NRAS* mutations co-existed in only one cell line (VOA-8862). Only one *BRAF* mutation was detected (D594G variant; VOA-6800 culture). Additionally we analyzed gene mutations and copy-number changes affecting 61 well-known MAPK-pathway genes is shown in Additional file 3: Table S2b. A summary of all *RAS* mutations, copy-number variation (CNV) findings, and MEKi sensitivity in each LGSC cell culture (n = 14) is shown in Table 1.

**Table 1:** Information of the LGSC cell cultures used in this study

Patient number	Sample name	Pathology at collection	Treatment status at collection	Cell culture type	% genome change by WES	Total number non-synonymous AA changes	KRAS/NRAS/BRAF mutation status	CNV changes affecting KRAS/NRAS/BRAF genes	Selumetinib IC50 (μM)	Trametinib IC50 (μM)	MEKi drug response
1	iOvCa241	Advanced LGSC	Post chemotherapy	Cell line	34	106	KRAS (G12D)	KRAS: allelic imbalance, CN gain	0.30	≤ 0.05	Sensitive
*2*	*VOA-1312*	*Advanced LGSC*	*Treatment naïve*	*Cell line*	*35*	*Undetermined*	*KRAS (G12V)* ^a^	*KRAS: allelic imbalance, CN gain*	*0.50*	*≤ 0.05*	*Sensitive*
3	VOA-8862	Advanced LGSC with MP	Treatment naïve	Cell line	66	24	KRAS (G12D), NRAS (C118Y)	KRAS: allelic imbalance, CN gain; NRAS: CN loss; BRAF: alellic imbalance, CN gain	5.00	≤ 0.05	Sensitive
*4*	*VOA-9164*	*Recurrent LGSC*	*Post anti-hormone therapy*	*Cell line*	*51*	*33*	*KRAS (G12V)*	*KRAS: allelic imbalance, CN gain; NRAS: CN loss; BRAF: high CN gain*	*0.30*	*≤ 0.05*	*Sensitive*
5	VOA-1056	Advanced MPSBT with invasive implants	Treatment naïve	Cell line	1	75	NRAS (Q61R)	NRAS: CN gain	0.30	≤ 0.05	Resistant
	VOA-3993	Recurrent LGSC	Post anti-hormone therapy	Cell line	16	84	NRAS (Q61R)	KRAS: allelic imbalance, CN gain; NRAS: CN gain	5.42	≤ 0.05	Resistant
*6*	*VOA-3448*	*Recurrent LGSC with MP*	*Post chemotherapy and anti-hormone therapy*	*Cell line*	*39*	*78*	*Wild type*	*BRAF: CN gain*	*0.58*	*≤ 0.05*	*Resistant*
	*VOA-3723*	*Recurrent LGSC with MP*	*Post chemotherapy and anti-hormone therapy*	*Cell line*	*40*	*96*	*Wild type*	*None*	*1.00*	*≤ 0.05*	*Resistant*
7	VOA-4627	Recurrent LGSC	Post chemotherapy, anti-hormone therapy, and targeted therapy	Cell line	56	111	Wild type	KRAS: CN gain; BRAF: LOH	8.75	0.08	Resistant
	VOA-4698	Recurrent LGSC	Post chemotherapy, anti-hormone therapy, and targeted therapy	Cell line	50	110	Wild type	KRAS: CN gain; NRAS: CN gain; BRAF: LOH	11.67	0.08	Resistant
*8*	*VOA-6406*	*Advanced LGSC*	*Post chemotherapy*	*Cell line*	*23*	*80*	*NRAS (Q61R)*	*NRAS: CN gain*	*0.64*	*≤ 0.05*	*Resistant*
9	VOA-6857	Recurrent LGSC	Post chemotherapy, anti-hormone therapy, and MEKi therapy	Transient culture	27	83	Wild type	None	n/a	n/a	n/a
*10*	*VOA-7604*	*Advanced LGSC with SBT*	*Treatment naïve*	*Transient culture*	*25*	*61*	*Wild type*	*KRAS: allelic imbalance, CN gain*	*n/a*	*n/a*	*n/a*
11	VOA-6800	Advanced LGSC	Post chemotherapy	Transient culture	29	68	BRAF (D594G)	None	n/a	n/a	n/a

The same typeface (roman or italic) has been used to help group cell lines from the same patient

*LGSC* low grade serous ovarian carcinoma, *MP* micropapillary, *MPSBT*micropapillary serous bordeline ovarian tumor, *SBT* serous borderline ovarian tumor, *WES* whole-exome sequencing analysis, *CNV* gene copy number variation, *AA* aminoacids, *IC50* 50% cell inhibitory concentration

^a^No buffy coat available; somatic from germline mutations are undiscernable

We found that all MEKi-Se cell lines (4/4; iOvCa241, VOA-1312, VOA-9164, VOA-8862) carried oncogenic mutations in *KRAS* (G12D or G12V), while MEKi-Re cell lines were either *NRAS* mutant (3/7; VOA-1056/VOA-3993 and VOA-6406), or *KRAS/NRAS* wt (4/7; VOA-3448/VOA-3723 and VOA-4627/VOA-4698). Of interest, the VOA-8862 cell line (mutations in both *KRAS* and *NRAS*) was found to be sensitive to all four MEKi tested. In this line, the *KRAS* mutation variant detected (G12D) is known to be oncogenic, while the *NRAS* mutation variant detected (C118Y) was not found in the COSMIC database, therefore its oncogenic potential remains unknown. We did not observe any obvious correlation between the degree of CNV in each cell line (copy number high versus low) and MEKi response.

### Proteomic differences between MEKi-Se and MEKi-Re LGSC cell lines

**Fig. 2:**
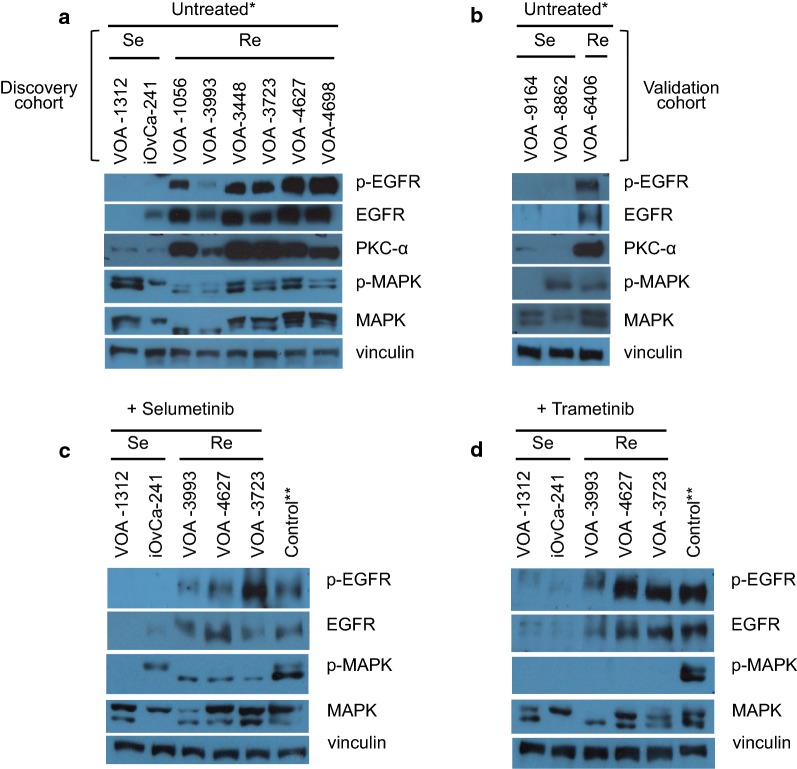
Differential expression of EGFR, p-EGFR and PKC-α between MEKi-Se and MEKi-Re LGSC cell lines by WB. **a** Confirmation of the RPPA results in untreated MEKi-Se and MEKi-Re lines (**a**, discovery cohort). EGFR, p-EGFR and PKC-α were increased in MEKi-Re lines (n = 5) compared to MEKi-Se lines (n = 2). **b** Validation of these protein biomarkers in three newly established LGSC cell lines classified according to their MEKi responsiveness (validation cohort). As found in the cell lines analyzed by RPPA, the new MEKi-Re line (n = 1) expressed higher levels of EGFR, p-EGFR and PKC-α compared to the two new MEKi-Se lines tested (n = 2). **c**, **d** Confirmation of RPPA results in MEKi treated cell lines. With MEKi treatment (selumetinib 1 μM and trametinib 0.1 μM) p-EGFR expression remained higher in MEKi-Re lines. As previously described, trametinib showed stronger inhibitory effects on MAPK (p-MAPK or p-ERK1/2) than selumetinib, even when used at ten times lower dose. (*) No DMSO. (**) Untreated VOA-4627 cells to control for drug inhibition effects on MAPK pathway

To identify biomarkers of MEKi response, we compared two MEKi-Se (VOA-1312, iOvCa241) and six MEKi-Re (VOA-1056/VOA-3993, VOA-3448/3723, VOA-4627/VOA-4698) LGSC cell lines using reverse phase protein array (RPPA) analysis. To do so, lines treated with DMSO, 1 μM selumetinib, or 0.1 μM trametinib were screened using a panel of 91 validated antibodies (see “Materials and methods” and Additional file 1: Table S1). We found 12 proteins that were differentially expressed between MEKi-Se and MEKi-Re cell lines (Additional file 4: Table S3). Among these proteins, EGFR and PKC-alpha were found to be overexpressed in all MEKi-Re lines independently of the treatment status. These two candidates were selected for validation and further study as they are regulators of MAPK signaling and play a role in MEKi and chemotherapy resistance in the literature [33–37]. WB analysis confirmed these findings (Fig. 2a), and also showed that p-EGFR (Y1068) was overexpressed in the MEKi-Re lines. Subsequently we subsequently validated the same candidates in the three newly established LGSC cell lines (VOA-6406, VOA-8862, VOA-9164) (see Fig. 2b). In keeping with the discovery cohort results, the MEKi-Re line (VOA-6406) expressed much higher levels of EGFR, p-EGFR and PKC-alpha than the two MEKi-Se lines (VOA-9164, VOA-8862) (Fig. 2b). As we previously described, p-MEK and p-ERK1/2 expression were not found to distinguish sensitive and resistant lines by WB [24].

We subsequently assessed differential protein expression by RPPA between MEKi-Se and MEKi-Re cell lines after selumetinib and trametinib treatment. Twenty-one and seventeen proteins were significantly different between MEKi-Se and MEKi-Re cells after selumetinib and trametinib treatment, respectively (Additional file 4: Table S3). Confirmation of the RPPA results was assessed by WB in one representative cell line from each individual patient (VOA-3993, VOA-4627, VOA-3723). Cell lines derived from the same patients at different time points in the disease course were not included for this analysis (VOA-1056, VOA-4698, VOA-3448). As seen in the untreated cells, WB confirmed increased p-EGFR levels in the MEKi-Re cell lines (Fig. 2c, d). As expected, trametinib more effectively inhibited MAPK phosphorylation than selumetinib. Differences in GSK3B and BID protein expression were also observed between MEKi-Se versus MEKi-Re cells by RPPA, however we were unable to validate these results using mass spectrometry (MS) analysis (data not shown). Interestingly, a number of the differentially expressed proteins (MEK-Se versus MEK-Re) were found to be drug-specific. These RPPA screening results are summarized in Additional file 4: Table S3, though these findings require further validation.

### In vitro evaluation of MEK and EGFR inhibition in MEKi-Re LGSC cell lines

To establish whether EGFR expression played a role in mediating MEKi resistance we evaluated the effects of EGFR inhibition (using erlotinib), with and without MEK inhibition (using selumetinib or trametinib), in four MEKi-Re LGSC cell lines (VOA-3723, VOA-3993, VOA-4627, and VOA-6406). IC50 values for erlotinib in these cell lines are shown in Additional file 5: Table S4. Except for VOA-3723, all MEKi-Re lines were highly resistant to single erlotinib treatment as observed in other ovarian cancer cell lines [38, 39]. Erlotinib doses chosen for the combined experiments are in keeping with erlotinib human serum levels [40]. Effects of single and combined drug treatment were evaluated using proliferation, viability, and WB assays. *EGFR* mutation and copy-number status were also evaluated. By WES, none of our LGSC cell lines carried activating mutations in *EGFR*, though some had copy-number changes affecting this gene. As summarized in Additional file 5: Table S4, we could not identify any obvious factors [EGFR CNV levels, levels of EGFR protein expression, phosphorylation, or sensitivity (IC50 values) to erlotinib treatment] that were associated with sensitivity to combination therapy.

**Fig. 3:**
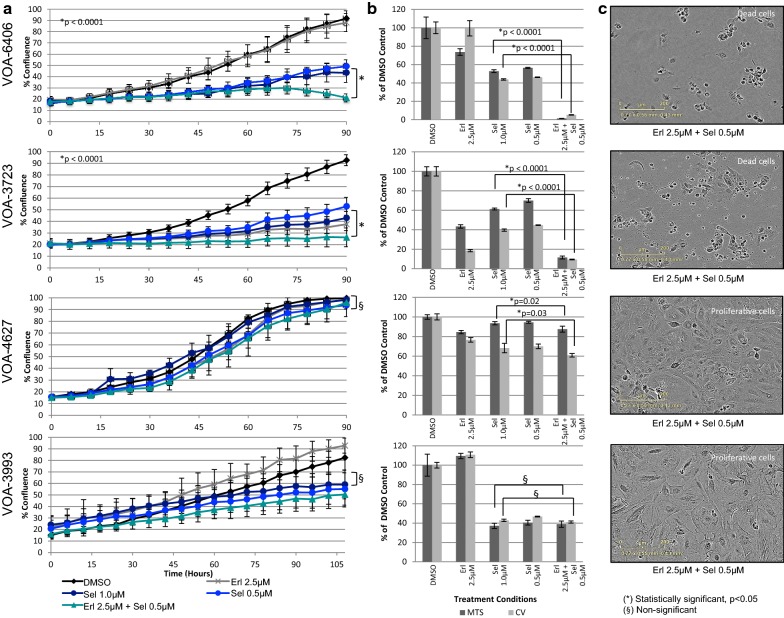
Effects of selumetinib and erlotinib single and combined drug treatments in four MEKi-Re LGSC cell lines. The graphed curves (**a**) represent the results from the proliferation experiments and the bar graphs (**b**) represent the results from the viability (MTS and CV) assays performed at the end of the proliferation experiments. The photomicrographs (**c**) show IncuCyte™ images at the completion of the experiment. All four cell lines were resistant to single selumetinib and erlotinib treatments. However, when the drugs were combined, VOA-6406 and VOA-3723 cell lines demonstrated complete cell death while VOA-4627 and VOA-3993 cells were shown to be resistant to the dual selumetinib and erlotinib treatment combination

With the highest dose of erlotinib treatment alone (2.5 μM, one dose, over 4–5 days), all four MEKi-Re cell lines continue to proliferate. Interestingly as shown in Fig. 3, a reduced dose of selumetinib (0.5 μM) in combination with erlotinib (2.5 μM) resulted in statistically significant decreases in cell proliferation and viability (p < 0.001; t-test) in 2 of the 4 cell lines tested (VOA-3723 and VOA-6406). At the end of these experiments, complete cell death of both cell lines was confirmed by image inspection on Incucyte™ (Fig. 3; and Additional file 6: Figure S2). Using Compusyn software analysis, synergistic drug effects (were demonstrable even using lower doses of erlotinib (1.25 or 0.63 μM) with selumetinib (0.5 μM) (Additional file 7: Table S5). These drug combinations were not effective in the other two lines tested (VOA-3993 and VOA-4627). Reduced dose of trametinib (0.05 μM) in combination with erlotinib (2.5 μM) resulted in similar results for the VOA-3723 cell line, but cytostatic effects for the VOA-6406 cell line. A summary of the synergistic drug effects are shown in Additional file 7: Table S5. Drug synergy was stronger with selumetinib and erlotinib combination than with trametinib and erlotinib combination.

**Fig. 4:**
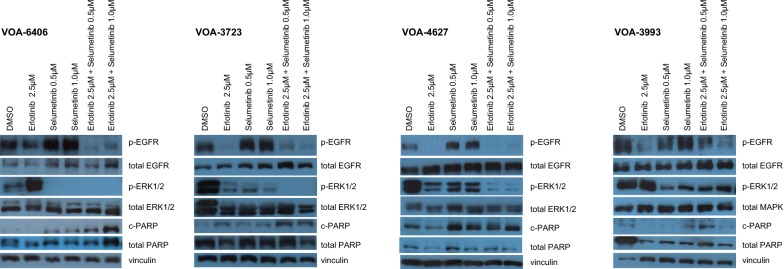
Cell signaling effects of selumetinib and erlotinib treatments in four MEKi-Re LGSC cell lines. As previously described, 24 h selumetinib treatment caused an increased in the levels of EGFR phosphorylation (p-EGFR Y1068) in 3 out of 4 MEKi-Re cell lines (VOA-6406, VOA-3723, and VOA-4627). As expected, erlotinib alone inhibited EGFR phosphorylation (p-EGFR Y1068) in all cell lines. Interestingly, erlotinib also inhibited MAPK phosphorylation (p-ERK1/2) in 2 out of 4 lines (VOA-3723 and VOA-4627), and increased it in another line (VOA-6406). No unique pathway interaction patterns for each of the MEKi-Re lines that were sensitive (VOA-6406, VOA-3723) or resistant (VOA-4627, VOA-3993) to erlotinib and selumetinib combination was detected

The effects of erlotinib, with and without MEKi treatment, on EGFR and MAPK signaling pathways were evaluated using WB. Levels of total and phosphorylated EGFR and ERK1/2, as well as total and cleaved PARP (c-PARP) were measured after 24 h treatment. Results from these experiments indicated that drug effects on cell signaling were cell line dependent (Fig. 4; and Additional file 8: Figure S3). As previously reported by our group, trametinib alone (0.1 μM) caused stronger inhibitory effects on ERK1/2 phosphorylation (p-ERK1/2) than selumetinib (1 μM). Unexpectedly, selumetinib treatment increased EGFR phosphorylation (p-EGFR Y1068) in 3 out of 4 MEKi-Re cell lines (VOA-6406, VOA-3723, and VOA-4627), however these effects were less obvious with trametinib treatment. As expected, erlotinib alone inhibited EGFR phosphorylation (p-EGFR Y1068) in all cell lines. Interestingly, erlotinib alone also inhibited ERK1/2 phosphorylation in 2 out of 4 lines (VOA-3723 and VOA-4627) and activated ERK1/2 phosphorylation in another line (VOA-6406). No pathway interaction was detected in the resistant VOA-3993 cell line. In these lines, while pathway interaction was observed, none of the changes in p-EGFR Y1068, p-ERK1/2 or c-PARP correlated with sensitivity or resistance to dual EGFRi and MEKi treatment. In the two MEKi-Re lines resistant to combination therapy (MEKi and erlotinib), the trametinib and erlotinib combination resulted in more apoptosis induction than the selumetinib and erlotinib combination (as measured by c-PARP).

### Effects of PKC-alpha inhibition in MEKi-Re LGSC cell lines

**Fig. 5:**
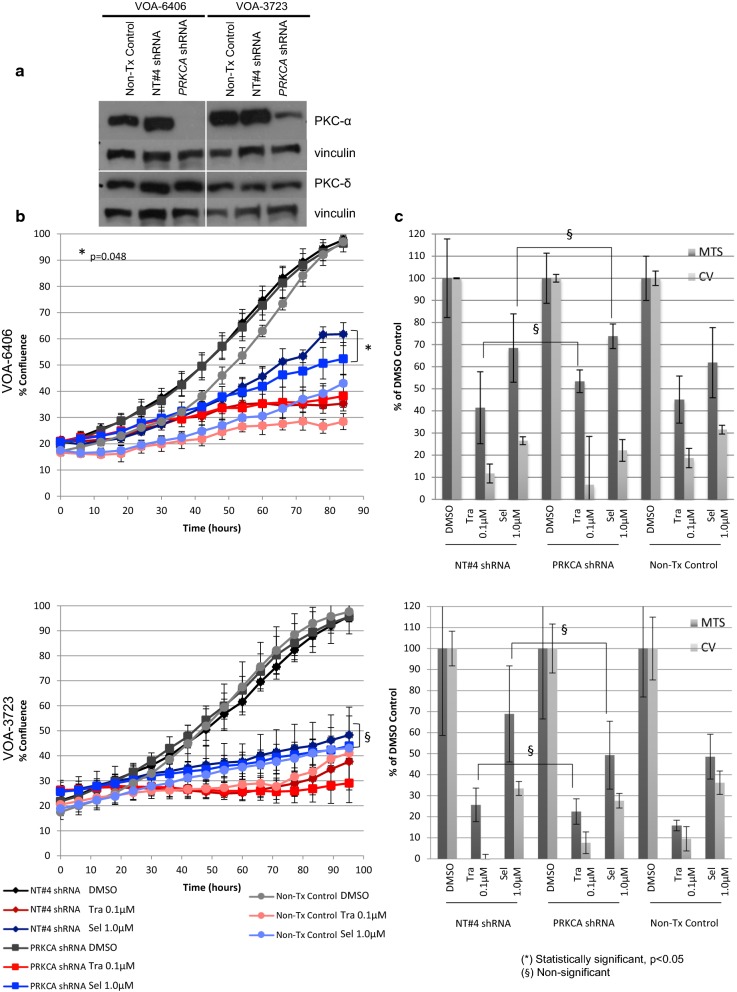
Effects of PRKCA knockdown in two MEKi-Re LGSC cell lines using lentiviral shRNA. **a** Determination of PKC-alpha (PKC-α) protein expression by WB. Transduction with lentiviral particles containing PRKCA shRNA resulted in a complete PKC-α protein knockdown in VOA-6406 cells and a partial knockdown in VOA-3723 cells. As detected by WES, VOA-3723 cells display PRCKA CN gain, which may explain the partial PKC-α protein knockdown. **b** Effects of PRKCA knockdown on cell proliferation. Reduction of PKC-α levels alone did not compromise cell proliferation in either of the two cell lines tested. Furthermore, PRKCA knockdown in combination with selumetinib treatment did not seem to significantly increase the sensitivity of these lines to selumetinib treatment. **c** Effects of PRKCA knockdown on cell viability (MTS and CV assays). As seen in these bar graphs, and similar to what we observed in the proliferation experiments, PRKCA knockdown did not seem to impact the viability of these lines

Genomic characterization of *PRKCA* by WES revealed that none of our LGSC cell lines carried activating mutations in *PRKCA*. It is interesting to note that two MEKi-Re cell cultures (VOA-3723 and VOA-6857) carried *PRKCA* copy-number gain and two MEKi-Se cells (VOA-9164 and VOA-8862) had *PRKCA* copy-number loss. To determine whether PKC-alpha protein expression played a role in mediating MEKi resistance we evaluated the effects of PKC-alpha knockdown using lentiviral shRNA, with and without selumetinib or trametinib in two MEKi-Re LGSC cell lines (VOA-6406 and VOA-3723). As shown in Fig. 5a, *PRKCA* shRNA resulted in a complete PKC-alpha protein knockdown in VOA-6406 cells and a partial knockdown in VOA-3723 cells by WB. Subsequent MEKi treatment (1.0 μM selumetinib or 0.1 μM trametinib) experiments showed no significant changes in cell viability when compared to non-target shRNA transduced lines (Fig. 5b). Proliferation assays demonstrated that PKC-alpha knockdown did not increase MEKi sensitivity in the VOA-3723 cells, but may slightly increase MEKi sensitivity to selumetinib in the VOA-6406 cell line (p = 0.048). This treatment combination was much less effective than EGFRi and MEKi combination in MEKi-Re LGSC cell lines as the cells continue to proliferate. Taken together, these results do not support PKC-alpha as a treatment target in LGSC to expand the efficacy of MEKi treatment.

## Discussion

Activating mutations affecting the MAPK pathway (RAS/RAF/MEK/ERK) are frequently found in cancer. MAPK pathway inhibitors, such as MEK inhibitors, were developed as targeted therapeutics to potentially treat such cancers [41, 42]. MEKi as single agents or in combination with other therapies have been studied for the treatment of melanoma, lung and colorectal cancers [43]. In 2013, the MEKi selumetinib was evaluated in a phase II clinical trial as a treatment for LGSC. Clinical responses (RECIST-1.1) to MEKi were observed in 15% of patients [22, 44]. While these responses were limited, response rates using conventional chemotherapy in patients with relapsed LGSC are disappointingly low (4%) [45]. More recently, a number of LGSC cases have been reported, highlighting dramatic and durable responses to MEKi treatment [22, 23, 46, 47]. Currently, there are no predictive biomarkers of MEKi response for LGSC. Identifying molecular markers which predict MEKi treatment efficacy will allow for pre-selection of patients who would benefit from this treatment, and avoid ineffective treatments and toxicities in those patients unlikely to respond.

In this study, we utilized genomic and proteomic techniques to molecularly characterize a collection of LGSC cell lines and primary cultures (derived from advanced/recurrent LGSC patients), and identify markers that predict response (sensitivity/resistance) to MEKi treatment in vitro. Genomic profiles of two of these cell models were compared with their corresponding tumor samples from the same patient and showed remarkably similar copy number profiles, supporting the utility of these cell models for preclinical research. Subsequent comparisons of genomic profiles from an additional twelve LGSC cell models showed frequent deletion of Chr9p (including loss of *MTAP* and *CDKN2A* genes) [48, 49] and oncogenic mutations in *KRAS* and *NRAS* genes, in agreement with results from previous studies on LGSC tumor tissues [13–15]. Additionally, *RAS* mutations were often associated with *RAS* copy number gain. As previously reported [24, 46, 50] we also detected multiple and distinct genomic alterations affecting other genes related to the MAPK cell signaling pathway. It is worth noting that the individual comparison of genomic profiles between LGSC cultures showed substantial variations in the types of gene mutations and copy-number alterations, indicating widespread molecular differences in LGSC tumors between patients.

Further evaluation of mutation profiles in eight LGSC cell lines with different sensitivity to MEKi treatment (two MEKi-Se and six MEKi-Re) showed oncogenic mutations in *KRAS* in all four MEKi-Se lines which were absent in all six MEK-Re lines. Previous results from a clinical trial using selumetinib (Farley et al. [22]) did not find a significant relationship between *RAS* mutation status and MEKi response rates in LGSC patients. It is important to note that tumor samples were not available for testing in 35% of the patients (18 of 52) in this study. In agreement with our results, two recent case reports on LGSC patients with remarkable and durable clinical responses (> 5 years) to MEKi therapy have reported oncogenic *KRAS* mutations (both G12V) in their tumors [23, 47]. As LGSC is often an indolent disease, the inclusion of patients with stable disease should also be considered in the future evaluation of *RAS* mutation status as a predictive biomarker. It is not unexpected that a single biomarker, such as *KRAS* mutation status, will not accurately predict responses to MEKi treatment, recognizing that LGSC harbor other MAPK-pathway gene mutations and significant MAPK copy number changes. Furthermore, KRAS copy-number amplification (described as one activating mechanism) could also play a role in mediating MEKi efficacy [44].

Using RPPA to compare MEKi-Se and MEKi-Re LGSC cell lines, we found that all MEKi-Re lines had higher levels of EGFR and PKC-alpha expression. These results were subsequently validated in three newly established LGSC cell lines. Using this approach, we also described proteomic changes specific to each MEKi tested (selumetinib or trametinib). The changes we observed may be particularly relevant when evaluating differences in drug efficacy, as MEKi may exhibit differences in MEK isoform specificity or off-target effects [24]. Interestingly, all MEKi-Re lines expressed higher levels of EGFR activation (p-EGFR Y1068) than the MEKi-Se lines. Although our study was limited to a small number of cell lines, we did not observe an obvious correlation between levels of EGFR and PKC-α protein expression and specific gene mutations or copy number changes in these genes.

In colorectal cancer, preclinical studies with BRAF inhibitors have reported adaptive feedback reactivation of MAPK signaling involving EGFR [33, 51]. This feedback signaling can be blocked by the addition of a MEKi. We similarly found evidence of MAPK feedback signaling following MEKi treatment that appears to play a role in MEKi resistances. Half of the MEKi-Re cells (2/4 cell lines) were effectively treated with selumetinib in combination with erlotinib, causing complete cell death. Combination therapy was effective in these two cell lines using drug doses that were below those that lacked efficacy as single drug treatments. Drug synergy was demonstrated using CompuSyn analyses in the two cell lines where cell death was demonstrated. In contrast, the other two lines tested continued to proliferate even with higher doses of the drug combination. We were unable to observe any obvious changes in p-EGFR and/or p-ERK that characterized the two combination-therapy resistant cell lines. As seen in our previous study [24], trametinib appeared to be a more effective inhibitor of ERK phosphorylation and cell proliferation than selumetinib. Based on its enhanced efficacy, it was more difficult to detect drug synergism using the erlotinib/trametinib combination than with the erlotinib/selumetinib combination.

There is a growing body of evidence supporting the use of combining a targeted therapy with other targeted agents or with traditional chemotherapeutic agents [29, 52]. Combination therapy using erlotinib and selumetinib was studied in a randomized phase II trial in lung cancer [53]. This drug combination did not prove to be effective in lung cancers irrespective of *KRAS* mutant status. Though the treatment was tolerated, significant side effects occurred with combination therapy. If these drug treatment combinations are going to be effective in LGSC, optimal drug dosing will be required in order to minimize side effects without loss of treatment efficacy.

Combination therapy with BRAFi and MEKi has remarkably improved survival in the adjuvant setting for patients with *BRAF* mutant melanomas, and combining a BRAFi and an EGFRi has improved tumor regression in *BRAF* mutant colorectal cancer xenografts [51, 54]. In a recent report, binimetinib in combination with paclitaxel was studied in platinum resistant ovarian cancer patients (NCT01649336). Two LGSC patients included in this trial showed response to this drug combination. These cases had also the largest reduction in target lesion size among the 25 ovarian cancer patients studied. MAPK pathway aberrations (*KRAS* G12D mutation and a *CUL1:BRAF* fusion) were identified in the tumors of both patients [44]. Additionally, two more LGSC patients with *KRAS* G12V [23, 47] and one with *MEK1* (Q56_V60del) gene mutations experienced disease stabilization in response to this drug treatment combination [46].

PKC-alpha expression has been implicated in chemotherapy drug resistance in some cancers [36, 37]. To explore its potential role in MEKi resistance, we inhibited PKC-α expression in two MEKi-Re lines. In the cell line where complete PKC-alpha protein knockdown was achieved, the effect of this treatment combination was not nearly as effective as combining MEKi and EGFRi. In the other line, where only partial knockdown of PKC-alpha protein expression was obtained, no changes in MEKi sensitivity were observed. Of interest, we found that this line contained *PRKCA* copy number gain. PKC-alpha knockdown by itself did not affect cell proliferation in either cell line. The results of our experiments suggest that PKC-alpha protein expression appears to be a predictive biomarker but is not a therapeutic target mediating MEKi resistance.

Identifying molecular characteristics to predict drug sensitivity/resistance in individual patients with solid tumors has proved to be challenging. The efficacy of therapies designed to target specific mutations are known to be dependent on the cancer type. For example, while BRAF inhibitors have shown to be effective in melanomas carrying *BRAF* mutations, they have demonstrated little effect in the treatment of *BRAF* mutant colon cancers [33, 55, 56]. In advanced LGSC, mutations in *KRAS* are more common than in *BRAF* [14, 19, 57, 58]. While MEKi have shown efficacy in some LGSC, still only a minority of patients respond to this treatment. Thus, it is of utmost importance to identify markers of drug treatment efficacy specific for each cancer type. A current clinical trial using the MEKi trametinib to treat patients with LGSC (NCT02101788), will include a translational research component in an attempt to identify predictive biomarkers in patient tumor samples.

## Conclusions

In summary, this proteogenomic study is the first to perform predictive biomarker discovery for MEKi treatment in LGSC cell lines. MEKi-Se cell lines were found to have oncogenic *KRAS* mutations and low levels of EGFR and PKC-alpha protein expression. The confirmation of these results in MEKi treated LGSC tumors samples could lead to better patient selection for MEKi treatment, and further avoid unnecessary treatment and toxicities in patients unlikely to respond. Our study also suggests that a significant portion of those LGSC patients whose tumors are unresponsive to MEKi therapy may benefit from combination therapy with EGFR and MEK inhibition. As LGSC xenografts are not yet available for research, we are currently unable to validate these results in vivo. However, we are now using our LGSC patient-derived cell lines to establish xenograft models. The potential predictive value of the three molecular markers of MEKi response identified in our LGSC cell line models should be considered for further validation in clinical trials using MEKi for the treatment of LGSC.

## Additional files

**Additional file 1: Table S1.** List of antibodies included in the RPPA analysis.

**Additional file 2: Figure S1.** Preclinical MEKi evaluation in novel LGSC cell lines.

**Additional file 3: Table S2.** List of all NsMs and genomic aberrations (CNV and NsMs) affecting 61 MAPK-related genes (WES analysis).

**Additional file 4: Table S3.** List of proteins differentially expressed between MEKi-se and MEKi-re LGSC cell lines (results from the RPPA and SPSS analysis).

**Additional file 5: Table S4.** EGFR information on LGSC cell lines.

**Additional file 6: Figure S2.** Biological effects of trametinib and erlotinib drug combination in MEKi-re LGSC cell lines.

**Additional file 7: Table S5.** Compusyn data from selumetinib/trametinib and erlotinib drug combinations in MEKi-re LGSC cell lines.

**Additional file 8: Figure S3.** Effects of trametinib and erlotinib combination on LGSC cell lines by WB analysis.

## Abbreviations

BCCA: British Columbia Cancer Agency
BCCF: British Columbia Cancer Foundation
c-PARP: cleaved PARP
Chr: chromosome
CI: combination index
CNA: copy-number aberration
CNV: copy-number variation
CV: crystal violet
dbSNP: single nucleotide polymorphism database
dFBS: defined fetal bovine serum
DMSO: dimethylsulfoxide
EGFRi: EGFR inhibitor
Erlo: erlotinib
GOC: Society of Gynecologic Oncology of Canada
HGSC: high-grade serous ovarian cancer/carcinoma
HRP: horse radish peroxidase
LGSC: low-grade serous ovarian cancer/carcinoma
MEKi-re: MEK inhibitor resistant
MEKi-se: MEK inhibitor sensitive
MEKi: MEK inhibitor
MTS: tetrazolium compound 3-(4,5-dimethylthiazol-2-yl)-5-(3-carboxymethoxyphenyl)-2-(4-sulfophenyl)-2H-tetrazolium
NsMs: non-synonymous mutation
OvCaRe: British Columbia Ovarian Cancer Research Program
p-EGFR: phosphorylated EGFR
p-ERK: phosphorylated ERK
p-MEK: phosphorylated MEK
PBS: phosphate saline buffer
PDX: patient-derived xenografts
PKC-α: PKC-alpha
POG: Personalized Oncogenomics Program
RPPA: reverse phase protein array
Sel: selumetinib
shRNA: short hairpin RNA
SNP: single nucleotide polymorphism
STR: short tandem repeat
Tra: trametinib
UBC: University of British Columbia
WB: western blot
WES: whole exome sequencing
WGS: whole genome sequencing
WB: western blot
p-val: p-value
