# Supplementary material for: Markers of MEK inhibitor resistance in low-grade serous ovarian cancer: EGFR is a potential therapeutic target
Source: Cancer Cell Int. 2019 Jan 8;19:10. doi: 10.1186/s12935-019-0725-1 (PMC6325847; doi:10.1186/s12935-019-0725-1)
Supplement: Supplementary file 2 — Additional file 2: Figure S1. Preclinical MEKi evaluation in novel LGSC cell lines. [file 12935_2019_725_MOESM2_ESM.pptx]

## Slide 1
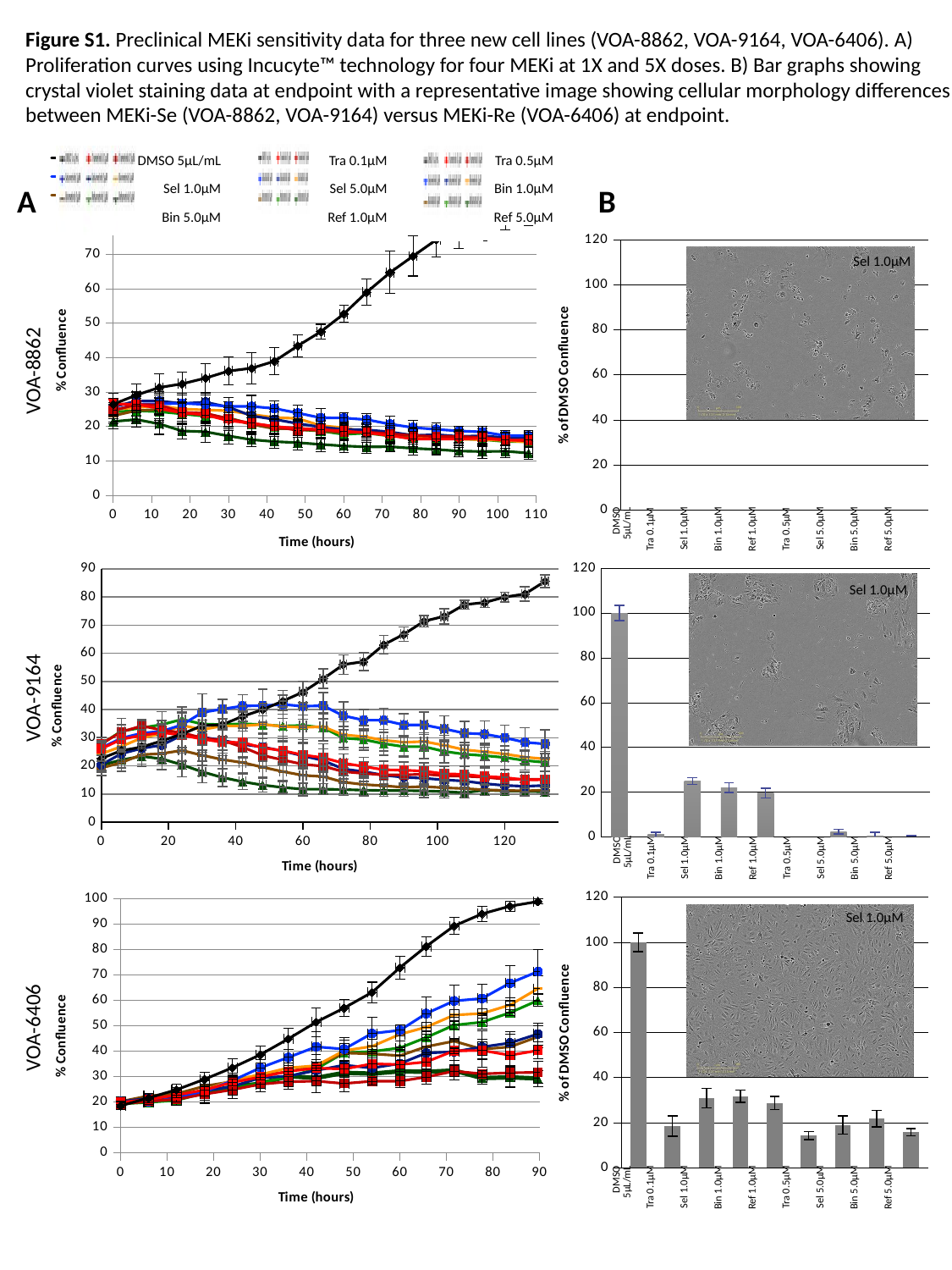

Figure S1. Preclinical MEKi sensitivity data for three new cell lines (VOA-8862, VOA-9164, VOA-6406). A) Proliferation curves using Incucyte™ technology for four MEKi at 1X and 5X doses. B) Bar graphs showing crystal violet staining data at endpoint with a representative image showing cellular morphology differences between MEKi-Se (VOA-8862, VOA-9164) versus MEKi-Re (VOA-6406) at endpoint.
### Chart
| Category | DMSO 5 uL/mL | Trametinib 0.1µM | Trametinib 0.5µM | Selumetinib 1µM | Selumetinib 5µM | Binimetinib 1µM | Binimetinib 5µM | Refametinib 1µM | Refametinib 5µM |
|---|---|---|---|---|---|---|---|---|---|| DMSO 5µL/mL | Tra 0.1µM | Tra 0.5µM |
| --- | --- | --- |
| Sel 1.0µM | Sel 5.0µM | Bin 1.0µM |
| Bin 5.0µM | Ref 1.0µM | Ref 5.0µM |
A
B
### Chart
| Category | CV |
|---|---|
| DMSO 5uL/mL | 100.0 |
| Trametinib 0.1µM | -3.714285714285716 |
| Selumetinib 1.0µM | 7.9642857142857135 |
| Binimetinib 1.0µM | 4.321428571428576 |
| Refametinib 1.0µM | 0.8928571428571459 |
| Trametinib 0.5µM | -4.357142857142855 |
| Selumetinib 5.0µM | -2.0 |
| Binimetinib 5.0µM | -2.0 |
| Refametinib 5.0µM | -1.8928571428571432 || | |
| --- | --- |
| | |
| | |
Sel 1.0µM
VOA-8862
| DMSO 5µL/mL | Tra 0.1µM | Sel 1.0µM | Bin 1.0µM | Ref 1.0µM | Tra 0.5µM | Sel 5.0µM | Bin 5.0µM | Ref 5.0µM |
| --- | --- | --- | --- | --- | --- | --- | --- | --- |
### Chart
| Category | DMSO 5 uL/mL, VOA9164 (1) 4K cells / well | TRA 0.1 µM, VOA9164 (1) 4K cells / well | TRA 0.5 µM, VOA9164 (1) 4K cells / well | SEL 1 µM, VOA9164 (1) 4K cells / well | SEL 5 µM, VOA9164 (1) 4K cells / well | BIN 1 µM, VOA9164 (1) 4K cells / well | BIN 5 µM, VOA9164 (1) 4K cells / well | REF 1 µM, VOA9164 (1) 4K cells / well | REF 5 µM, VOA9164 (1) 4K cells / well |
|---|---|---|---|---|---|---|---|---|---|
### Chart
| Category | |
|---|---|
Sel 1.0µM
VOA-9164
| DMSO 5µL/mL | Tra 0.1µM | Sel 1.0µM | Bin 1.0µM | Ref 1.0µM | Tra 0.5µM | Sel 5.0µM | Bin 5.0µM | Ref 5.0µM |
| --- | --- | --- | --- | --- | --- | --- | --- | --- |
### Chart
| Category | CV |
|---|---|
| DMSO 5µL/mL | 100.0 |
| Trametinib 0.1µM | 18.506998444790064 |
| Selumetinib 1.0µM | 30.94867807153962 |
| Binimetinib 1.0µM | 31.778123379989616 |
| Refametinib 1.0µM | 28.73682391567306 |
| Trametinib 0.5µM | 14.35977190254018 |
| Selumetinib 5.0µM | 18.990841541385834 |
| Binimetinib 5.0µM | 21.824779678589945 |
| Refametinib 5.0µM | 15.742180749956798 |
### Chart
| Category | DMSO 5 uL/mL | Trametinib 0.1µM | Trametinib 0.5µM | Selumetinib 1µM | Selumetinib 5µM | Binimetinib 1µM | Binimetinib 5µM | Refametinib 1µM | Refametinib 5µM |
|---|---|---|---|---|---|---|---|---|---|Sel 1.0µM
VOA-6406
| DMSO 5µL/mL | Tra 0.1µM | Sel 1.0µM | Bin 1.0µM | Ref 1.0µM | Tra 0.5µM | Sel 5.0µM | Bin 5.0µM | Ref 5.0µM |
| --- | --- | --- | --- | --- | --- | --- | --- | --- |
