# Supplementary material for: Markers of MEK inhibitor resistance in low-grade serous ovarian cancer: EGFR is a potential therapeutic target
Source: Cancer Cell Int. 2019 Jan 8;19:10. doi: 10.1186/s12935-019-0725-1 (PMC6325847; doi:10.1186/s12935-019-0725-1)
Supplement: Supplementary file 6 — Additional file 6: Figure S2. Biological effects of trametinib and erlotinib drug combination in MEKi-re LGSC cell lines. [file 12935_2019_725_MOESM6_ESM.pptx]

## Slide 1
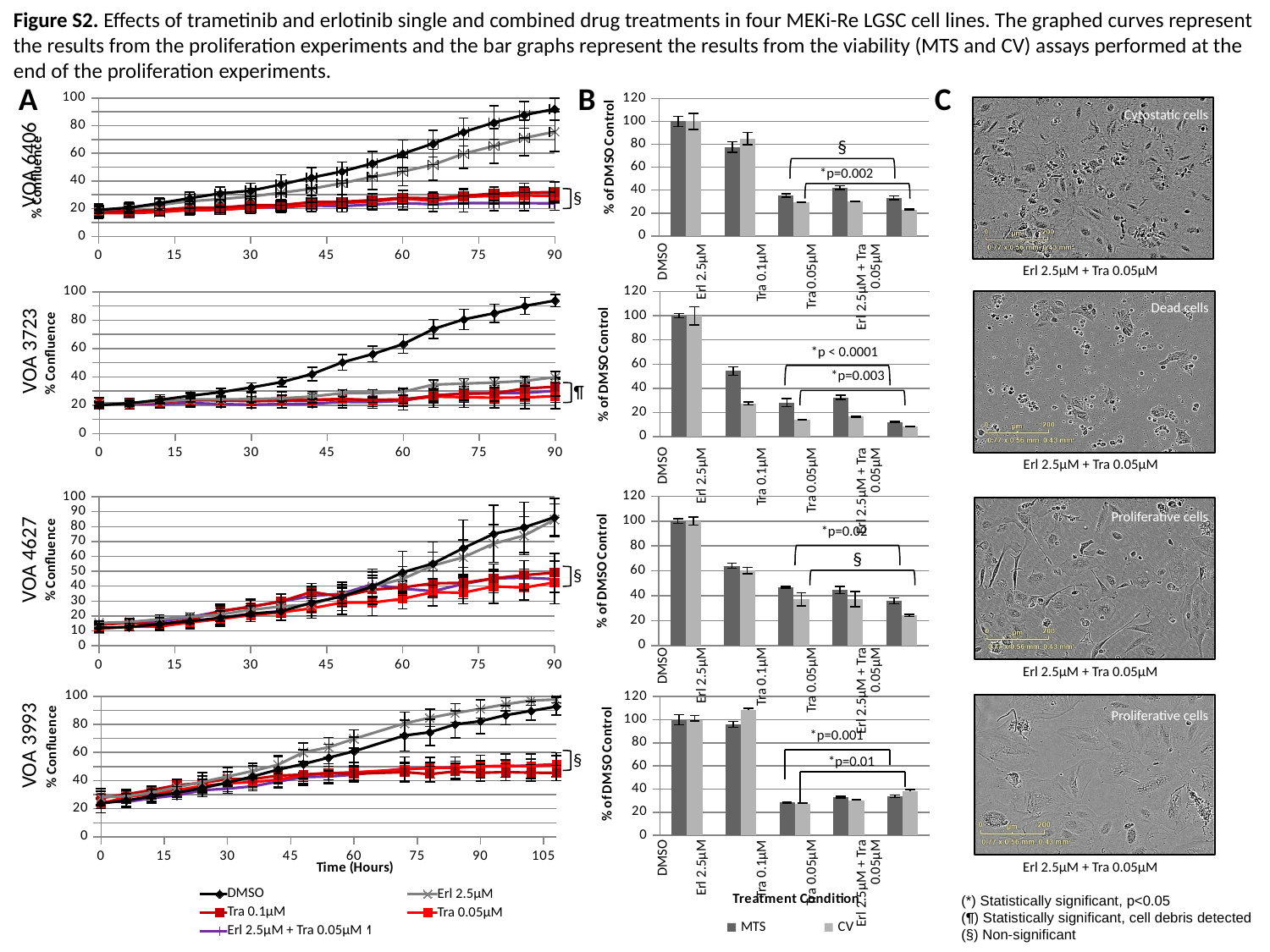

Figure S2. Effects of trametinib and erlotinib single and combined drug treatments in four MEKi-Re LGSC cell lines. The graphed curves represent the results from the proliferation experiments and the bar graphs represent the results from the viability (MTS and CV) assays performed at the end of the proliferation experiments.
### Chart
| Category | MTS | CV |
|---|---|---|
| _x0004_DMSO | 100.0 | 100.0 |
| _x000f_Erlotinib 2.5µM | 77.60736196319019 | 85.01742160278748 |
| _x0010_Trametinib 100nM | 35.36371603856267 | 29.50058072009293 |
| _x000f_Trametinib 50nM | 42.17791411042945 | 30.42973286875723 |
| _x0014_Erl 2.5µM + Tra 50nM | 33.23838737949168 | 22.99651567944252 |
### Chart
| Category | | Erlotinib 2.5µM | Trametinib 100 nM | Trametinib 50 nM | |
|---|---|---|---|---|---|A
B
C
Cytostatic cells
§
VOA 6406
*p=0.002
§
| DMSO | Erl 2.5µM | Tra 0.1µM | Tra 0.05µM | Erl 2.5µM + Tra 0.05µM |
| --- | --- | --- | --- | --- |
### Chart
| Category | | Erlotinib 2.5µM | Trametinib 100 nM | Trametinib 50 nM | |
|---|---|---|---|---|---|Erl 2.5µM + Tra 0.05µM
### Chart
| Category | | |
|---|---|---|
| _x0004_DMSO | 100.0 | 100.0 |
| _x000f_Erlotinib 2.5µM | 54.3105909220668 | 27.509293680297386 |
| _x0010_Trametinib 100nM | 28.2543534113617 | 13.777881040892188 |
| _x000f_Trametinib 50nM | 32.40793605481015 | 16.496282527881018 |
| _x0014_Erl 2.5µM + Tra 50nM | 12.13245789323437 | 8.410780669144975 |
Dead cells
VOA 3723
*p < 0.0001
*p=0.003
¶
### Chart
| Category | MTS | CV |
|---|---|---|
| _x0004_DMSO | 100.0 | 100.0 |
| _x000f_Erlotinib 2.5µM | 64.1122098022356 | 60.28797027403622 |
| _x0010_Trametinib 100nM | 46.70034393809116 | 37.01811425917323 |
| _x000f_Trametinib 50nM | 44.507738607050726 | 37.157454714352035 |
| _x0014_Erl 2.5µM + Tra 50nM | 35.995270851246765 | 24.198792382721752 || DMSO | Erl 2.5µM | Tra 0.1µM | Tra 0.05µM | Erl 2.5µM + Tra 0.05µM |
| --- | --- | --- | --- | --- |
Erl 2.5µM + Tra 0.05µM
### Chart
| Category | | Erlotinib 2.5 µM | Trametinib 100 nM | Trametinib 50 nM | |
|---|---|---|---|---|---|
Proliferative cells
*p=0.02
VOA 4627
§
§
### Chart
| Category | MTS | CV |
|---|---|---|
| _x0004_DMSO | 100.0 | 100.0 |
| _x000f_Erlotinib 2.5µM | 96.02500509614731 | 108.39378238341968 |
| _x0010_Trametinib 100nM | 28.36855337364952 | 26.943005181347147 |
| _x000f_Trametinib 50nM | 33.138547258272745 | 30.0518134715026 |
| _x0014_Erl 2.5µM + Tra 50nM | 33.77046952503905 | 38.13471502590673 |
### Chart
| Category | | Erlotinib 2.5 µM | Trametinib 100 nM | Trametinib 50 nM | |
|---|---|---|---|---|---|| DMSO | Erl 2.5µM | Tra 0.1µM | Tra 0.05µM | Erl 2.5µM + Tra 0.05µM |
| --- | --- | --- | --- | --- |
Erl 2.5µM + Tra 0.05µM
Proliferative cells
*p=0.001
VOA 3993
§
*p=0.01
| DMSO | Erl 2.5µM | Tra 0.1µM | Tra 0.05µM | Erl 2.5µM + Tra 0.05µM |
| --- | --- | --- | --- | --- |
Erl 2.5µM + Tra 0.05µM
DMSO
Tra 0.1µM
Erl 2.5µM + Tra 0.05µM
Erl 2.5µM
Tra 0.05µM
(*) Statistically significant, p<0.05
(¶) Statistically significant, cell debris detected
(§) Non-significant
