# Supplementary material for: Markers of MEK inhibitor resistance in low-grade serous ovarian cancer: EGFR is a potential therapeutic target
Source: Cancer Cell Int. 2019 Jan 8;19:10. doi: 10.1186/s12935-019-0725-1 (PMC6325847; doi:10.1186/s12935-019-0725-1)
Supplement: Supplementary file 8 — Additional file 8: Figure S3. Effects of trametinib and erlotinib combination on LGSC cell lines by WB analysis. [file 12935_2019_725_MOESM8_ESM.pptx]

## Slide 1
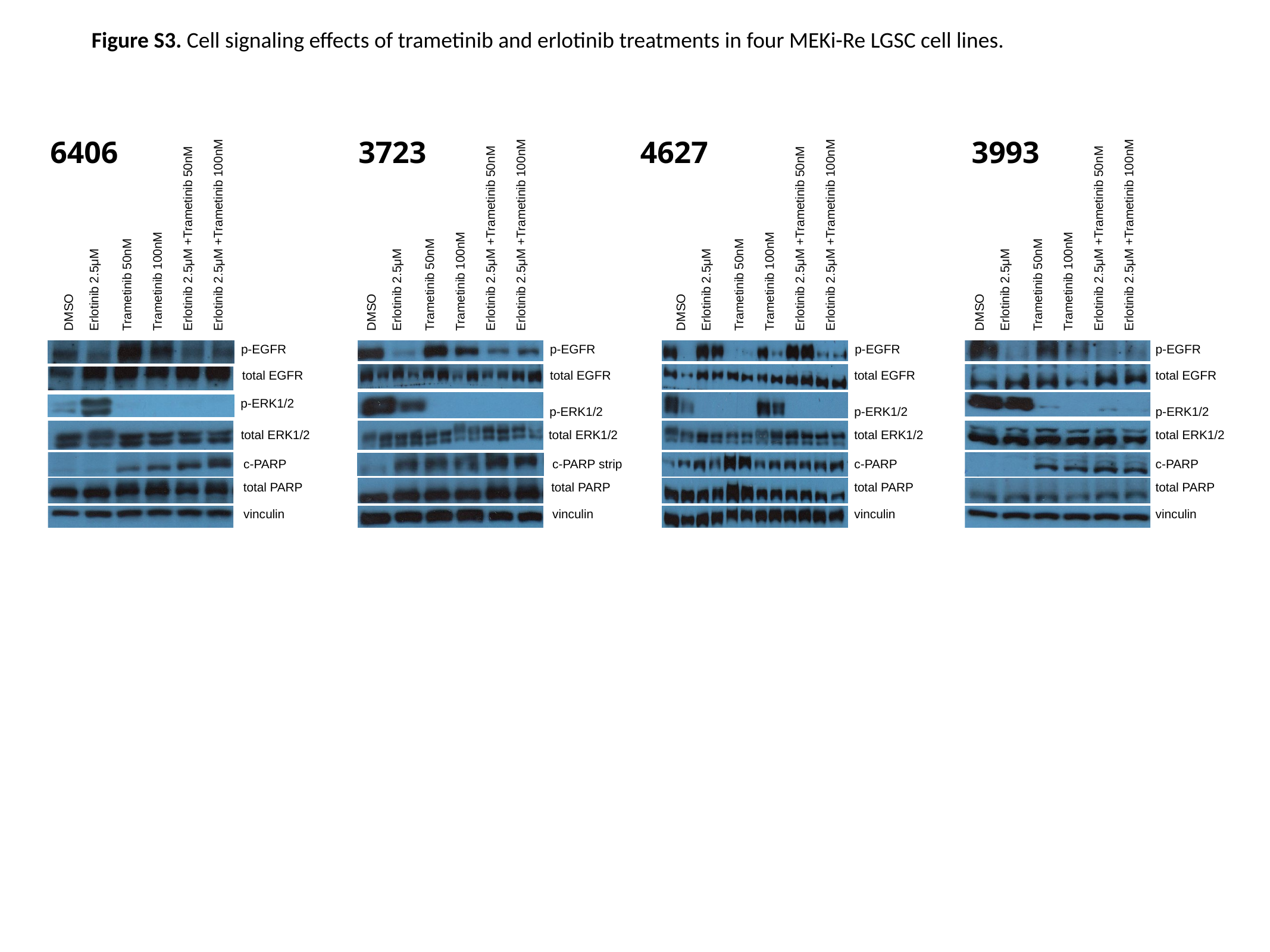

Figure S3. Cell signaling effects of trametinib and erlotinib treatments in four MEKi-Re LGSC cell lines.
6406
3723
4627
3993
Erlotinib 2.5μM +Trametinib 100nM
Erlotinib 2.5μM +Trametinib 50nM
Trametinib 50nM
Trametinib 100nM
Erlotinib 2.5μM
DMSO
Erlotinib 2.5μM +Trametinib 100nM
Erlotinib 2.5μM +Trametinib 50nM
Trametinib 50nM
Trametinib 100nM
Erlotinib 2.5μM
DMSO
Erlotinib 2.5μM +Trametinib 100nM
Erlotinib 2.5μM +Trametinib 50nM
Trametinib 50nM
Trametinib 100nM
Erlotinib 2.5μM
DMSO
Erlotinib 2.5μM +Trametinib 100nM
Erlotinib 2.5μM +Trametinib 50nM
Trametinib 50nM
Trametinib 100nM
Erlotinib 2.5μM
DMSO
p-EGFR
p-EGFR
p-EGFR
p-EGFR
total EGFR
total EGFR
total EGFR
total EGFR
p-ERK1/2
p-ERK1/2
p-ERK1/2
p-ERK1/2
total ERK1/2
total ERK1/2
total ERK1/2
total ERK1/2
c-PARP
c-PARP strip
c-PARP
c-PARP
total PARP
total PARP
total PARP
total PARP
vinculin
vinculin
vinculin
vinculin
